# Dental and Microbiological Risk Factors for Hospital-Acquired Pneumonia in Non-Ventilated Older Patients

**DOI:** 10.1371/journal.pone.0123622

**Published:** 2015-04-29

**Authors:** Victoria C. Ewan, Andrew D. Sails, Angus W. G. Walls, Steven Rushton, Julia L. Newton

**Affiliations:** 1 Newcastle University Institute for Ageing, Newcastle upon Tyne, United Kingdom; 2 Public Health England, Microbiology Services, Newcastle Laboratory, Newcastle upon Tyne, United Kingdom; 3 Edinburgh Dental Institute, University of Edinburgh, Edinburgh, United States of America; 4 Biological Modelling, Newcastle University, Newcastle upon Tyne, United Kingdom; University of Dundee, UNITED KINGDOM

## Abstract

Hospital acquired pneumonia (HAP) is often fatal in older patients. The mouth is the main reservoir of infection and studies have suggested that oral hygiene interventions may prevent HAP. The aim of this study was to investigate associations between HAP and preceding a) heavy dental plaque and b) oral carriage of potential respiratory pathogens in older patients with lower limb fracture to determine the target for intervention studies.

**Methods:**

We obtained a time series of tongue/throat swabs from 90 patients with lower limb fracture, aged 65-101 in a general hospital in the North East of England between April 2009-July 2010. We used novel real-time multiplex PCR assays to detect *S*. *aureus*, MRSA, *E*. *coli*, *P*. *aeruginosa*, *S*. *pneumoniae*, *H*. *influenza* and *Acinetobacter* spp. We collected data on dental/denture plaque (modified Quigley-Hein index) and outcomes of clinician-diagnosed HAP.

**Results:**

The crude incidence of HAP was 10% (n = 90), with mortality of 80% at 90 days post discharge. 50% of cases occurred within the first 25 days. HAP was not associated with being dentate, tooth number, or heavy dental/denture plaque. HAP was associated with prior oral carriage with *E*. *coli/S*. *aureus/P*.*aeruginosa*/MRSA (p = 0.002, OR 9.48 95% CI 2.28-38.78). The incidence of HAP in those with carriage was 35% (4% without), with relative risk 6.44 (95% CI 2.04-20.34, p = 0.002). HAP was associated with increased length of stay (Fishers exact test, p=0.01), with mean 30 excess days (range -11.5-115). Target organisms were first detected within 72 hours of admission in 90% participants, but HAP was significantly associated with *S*. *aureus*/MRSA/*P*. *aeruginosa/E*. *coli* being detected at days 5 (OR 4.39, 95%CI1.73-11.16) or 14 (OR 6.69, 95%CI 2.40-18.60).

**Conclusions:**

Patients with lower limb fracture who were colonised orally with *E*. *coli/ S*. *aureus*/MRSA/*P*. *aeruginosa* after 5 days in hospital were at significantly greater risk of HAP (p = 0.002).

## Introduction

Hospital acquired pneumonia (HAP) is now the most common hospital associated infection in England[1] and one of the most common complications following lower limb fracture in older adults, (incidence of 8.6–10%, [2–4] mortality of 12–43% [2, 5–7]). Efforts to prevent HAP are important because of the associated high mortality, hospital costs, functional decline and increased length of stay [8–10]. HAP appears to arise from interactions between three main risk factor groups: resident oral microbiota, aspiration potential (dysphagia, reduced conscious level) and host factors (age, frailty, comorbidity); the first is the most easily modifiable, despite not having the strongest effects. However few studies include non-ventilated, frail older patients because of difficulties with diagnosis and recruitment, despite these patients making up the majority of HAP cases.

The mouth is the main reservoir of infection, and matching organisms (>95% similarity by pulse-field gel electrophoresis) have been detected in dental plaque and bronchoalveolar lavage fluids in patients with ventilator associated pneumonia (VAP) [11], implicating aspiration of organisms within dental plaque as the cause of the pneumonia. In addition, aspiration pneumonia was reported to be associated with increased number of teeth or decayed teeth, presence of *Staphylococcus aureus* in saliva and *Porphyromonas gingivalis* in dental plaque in dentate patients [12]. It is therefore possible that dental plaque, a removable matrix rich with oral bacteria which is a pre-requisite for caries, is the common driver. Dental plaque has been implicated as a reservoir for potential respiratory pathogens not usually native to the mouth[11, 13–16], such as Enterobactericeae. Interventions to reduce the oral bioburden and thus pneumonia have been successfully trialled in ventilated patients [17–19], and combined with professional dental hygienist intervention, with moderate success in nursing home residents [20, 21]. An intervention trial of tooth-brushing up to four times daily resulted in a 37% reduction in cases of HAP, with cost savings of $1.6 million in avoided antibiotics and bed days [22].

However, oral hygiene interventions also successfully decreased febrile days in edentate (no teeth) patients in nursing homes in Japan [20], and similar rates of pneumonia have been observed regardless of whether dentate or edentate [20, 23]. In addition, *S*. *aureus* and coliform bacteria were most often found in saliva and soft tissue [24], and colonisation with respiratory pathogens correlated poorly with heavier dental plaque in other studies [14, 16]. Given that the majority of culture-positive HAP has been aetiologically linked with non-dental organisms such as *Escherichia coli*, *S*. *aureus* etc.[25], the relative contributions of these organisms versus dental plaque associated organisms is unclear. In addition, some oral hygiene intervention studies in ventilated patients have produced negative results [26, 27].

While numerous studies linking VAP with oropharyngeal colonisation with respiratory studies have been conducted, few studies have linked non-ventilated HAP with prior oropharyngeal colonisation [28–31]. Of these, one was a case-control study[28], one was of lung cancer patients undergoing operative treatment [30], another of upper abdominal surgical patients [31], and the fourth was a ten year follow up study of limited baseline data, which did consider dentition [29]. All of these studies used culture to determine colonisation, and took samples at one-two time points.

In order to design a robust oral hygiene intervention it was important to clarify the relative importance of oropharyngeal colonisation (and which organisms therein were important), dentition and dental/denture plaque to the development of HAP in older patients, and understand how these potential risk factors interacted. In addition no studies had used molecular methods to detect bacteria, nor taken time series of samples to determine the effects of hospitalisation on the oral flora.

The aim of this study was to investigate whether HAP was associated with a) prior oral carriage of respiratory pathogens or b) prior heavy dental or denture plaque. We included old, frail and cognitively impaired patients as these are the characteristics of the majority of patients in hospital. We chose patients with hip or other lower limb fracture because, for most, operative intervention is mandatory, and reducing post-operative pneumonia in this group is therefore unarguable despite frailty, advanced age or impaired cognition. Given that bronchoscopy would have been inappropriate in many patients, we used clinician diagnosis of HAP, supported by American Thoracic Society guidelines.

The main aim of the study was to determine whether HAP was commoner in patients whose mouths had acquired or become colonised by organisms detectable by the PCR panel within 14 days of hospital admission.

## Materials and Methods

Patients with lower limb fracture were recruited prospectively from the orthopaedic wards at Newcastle General Hospital. We took oral samples (tongue and throat) at five time-points during the admission (1, 3, 5, 7 and 14 days after admission). We also undertook three dental examinations to assess tooth number and dental/denture plaque 1, 7 and 14 days after admission. Frailty indices were calculated and demographic data were recorded at the first visit. Patients were followed up thrice weekly until discharge to ascertain cases of pneumonia, and case notes were reviewed weekly. Follow-up telephone calls to patient and General practitioner were made at 90 days. During study recruitment, we developed novel real-time PCR assays to characterise the oral colonisation dynamics of seven major respiratory bacterial pathogens. Anonymised oral samples were then analysed using the real-time multiplex PCR assays after recruitment had terminated. We then prospectively investigated associations between incidence of a) infection events (HAP), and b) colonisation events by real-time PCR, dental, microbiological and medical risk factors. We used Fisher’s exact test and univariate generalised linear models for the former and multivariate generalised linear models for the latter. Further details are given below.

### Patient recruitment and consent

Patients were identified at the daily trauma meeting at Newcastle General Hospital between April 2009-July 2010. Recruitment occurred pre-operatively where possible, or on the first post-operative day otherwise.

Inclusion criteria were age >65 and lower limb fracture. Exclusion criteria were immunosuppression within last three months (immunosuppressive drugs, chemotherapy or radiotherapy or > = 10mg oral prednisolone but not inhaled steroids), acute illness, palliative care and community acquired pneumonia. We excluded patients with acute illness because operative treatment was delayed in this group due to urgent medical therapy being given. Assuming that perioperative antibiotics, anaesthetic with laryngeal mask airway, and operation might affect the oral flora, it would be difficult to compare acutely unwell patients with those who were operated upon within 48 hours of admission. Immunosuppression was an exclusion criterion because the immune system is likely to play an important role in determining membership and diversity of the oral microbiota, and ought to studied separately. The sample was therefore biased towards “well” patients.

A power calculation, based on a presumed exposure (oral colonisation) of 20% and outcome incidence of HAP of 10% in unexposed at 80% power and the 0.05 significance level suggested that recruiting 200 patients would be able to detect a 20% difference in the incidence of HAP between colonised and uncolonised persons [32]. An exposure of 20% was based on two previous papers [33, 34], and the incidence of HAP was based on 9% incidence found by Roche et al. [2].

### Ethics statement

Ethical approval was granted by the Newcastle and North Tyneside 2 research ethics committee, which had a special interest in adults lacking capacity. The research was conducted as per the Mental Capacity Act 2005 (United Kingdom) guidelines, and every effort was made to include all persons with cognitive impairment because these are the very people that hospital acquired pneumonia affect. To exclude these persons would mean that the research outcomes would be less useful when applying results to real life populations. In addition, the taking of oral swabs was deemed minimally intrusive and wholly without risk to participants.

Written patient consent was obtained. Where there were concerns regarding capacity, a capacity assessment was undertaken by VE (Could the patient take in, believe, weigh the information and come to a reasoned judgement?). All efforts were made to allow patients to make their own decision, including repeating information, visits at different times and finding visual or hearing aids. If the patient was found not to have capacity, then a relative (personal consultee) was sought and invited to provide written consent on the patient’s behalf, taking into account what they knew of their relative’s beliefs, wishes and condition. If the patient had no relatives then a professional independent of the study was sought (professional consultee), and invited to provide written consent, again taking into account the patient’s condition. According to the Act, a researcher may nominate another individual who is not connected to the project according to local guidelines to act on the participant's behalf. In this study, one patient fell into this category, and a qualified nurse who had been caring for that patient acted on their behalf. No power of attorney was assigned. The nurse was unconnected with the study and its personnel, and had not previously worked together. If any patient showed any signs of not wishing to take part in the study (for example, closing mouth to swabs or appearing unhappy or distressed) they were immediately withdrawn, even if written consent had been provided on their behalf.

### Routine Care

All patients (apart from two treated without operation) received peri-operative antibiotics (three doses of cefuroxime 750mg 12 hourly until August 2009, three doses of teicoplanin 400mg 12 hourly thereafter). All patients received 4500 international units of tinzaparin subcutaneously, unless already anticoagulated on warfarin. Routine postoperative analgesia was co-codamol 30/500mg four times daily. Patients were routinely screened for MRSA and decolonised with chlorhexidine mouthwash and antibacterial toothpaste if found positive. No specific oral hygiene policy was in operation at the time of the study and the study team did not undertake any oral hygiene intervention. Patients relied on nursing staff helping with oral hygiene and bringing equipment to their beds if unable to mobilise, and leaving equipment within their reach. Patients with dentures were given denture pots if these were available, but these did not necessarily contain fluid.

### Recording of demographic variables

We recorded demographic data including age, residence, gender, weight, comorbidity, and prescribed drugs. We calculated functional scores, including the Barthel index (0–20, with 20 meaning needs no help with activities of daily living) [35], the Clinical Frailty scale (1–9, 1 being the fittest, increasing with frailty) [36] and the Hierarchical Assessment of Balance and Mobility (HABAM) score (1–63, higher score meaning better mobility)[37]. We also calculated Charlson comorbidity indices (www.medal.org) for each participant. All data were entered into a Microsoft Access database (VE). Complications were recorded prospectively from case notes review. Aspiration episodes were noted opportunistically (i.e. choking episode witnessed by research team while giving patient a drink during sample collection) rather than systematically, and are included for interest.

### Recording of oral hygiene variables

The number of teeth or teeth on dentures was recorded. Dental and denture plaque (full mouth) were scored by the bedside at days 1, 7 and 14 using the modified Quigley Hein index (VE) [38, 39]. Scores of 0–5 were recorded for each tooth surface (2 per tooth) with 0 meaning no visible plaque and 5 meaning over 2/3 of tooth covered in plaque. Midway through the study, intra-rater calibration using 138 surfaces gave kappa scores of 80.9% (good). Dentures in use were removed for scoring, and were not cleaned. Dentures not in use were not scored. In order to create a single plaque score per patient, a quartile score was assigned to modified Quigley Hein indices (both dental and denture), and where scores were discordant, the higher score was used (disclosing tablets could not be used and therefore scores may have been under-estimated).

### Collection of oral samples

Flocked swabs were used to sample the tongue and throat at days 1, 3, 5, 7, and 14 (or nearest working day) between 8.30am-12 pm, or 1-4pm. No special instructions were given to patients prior to sample collection.

Throat swabs were taken from anterior faucial pillars, using a back-and-forth motion three times. Tongue swabs were taken by making three strokes posteriorly-anteriorly, then rotating the swab 180° and making a further three strokes. Swab tips were transferred into 2ml microtubes, transported to the Health Protection Agency (HPA) within four hours of collection, and stored at 2–8°C until DNA extraction within 48 hours. Samples were anonymised and stored at -80°C after DNA extraction.

### Diagnosis of HAP and HAP/LRTI

HAP was recorded when antibiotics were started for pneumonia by the responsible clinician after 48 hours in hospital (that is, a clinical endpoint). The diagnosis was further characterised using American Thoracic Society (ATS) [40] and British Society for Antimicrobial Chemotherapy (BSAC) guidelines [41], both of which required a chest radiograph with new infiltrates. ATS guidelines also require two of fever >38°C, leucocytosis or leucopoenia and purulent secretions. BSAC guidance suggests purulent tracheal secretions and new/persistent infiltrates on chest radiograph, increased oxygen requirement, leucocytosis >10,000 /mm^3^ or <4,000/mm^3^, and core temperature >38.3°C can be used to identify those who would benefit from antibiotics. It should be noted that these criteria were validated on ventilated patients. Episodes of lower respiratory tract infection (LRTI) were also noted, and were diagnosed by clinician initiated antibiotics for productive cough in the absence of positive chest x-ray findings. If a patient was commenced on intravenous antibiotics for productive cough but died of this illness without a chest x ray, this was considered to be pneumonia.

### Real-time PCR analysis

Anonymised samples were analysed using multiplex real-time PCR assays after patient follow-up was complete (September 2010-December 2011) by a single clinical scientist (GE). The assays detected *S*. *aureus*, Meticillin resistant *S*. *aureus* (MRSA), *E*. *coli*, *Pseudomonas*. *aeruginosa*, *Streptococcus pneumoniae*, *Haemophilus influenzae*, *Acinetobacter* spp. and the *gap* gene (human housekeeping gene). The five-well multiplex real-time PCR assays were designed in conjunction with the Health Protection Agency Newcastle for this study (unpublished, *S*. *aureus* and MRSA and *gap* assays taken from existing literature [42, 43]), and the primers and probe sequences used in these assays are shown in S1 Table.

Real time PCR assays were carried out in 50μl volumes and contained 25μl Universal PCR master mix (Applied Biosystems, Warrington, UK), primers (final concentration 20μM), probe (final concentration 10μM), 5μl of DNA template and PCR grade water. Thermal cycling and data analysis were conducted on two Taqman 7500 instruments (Applied Biosystems). Thermal cycling conditions were as follows: 50°C for 2 minutes, 95°C for 10 minutes, then 40 cycles of 95°C for 15s then 60°C for 1 min.

The assays were successfully validated on 63 previously identified clinical culture isolates of target and oral bacteria (sensitivity 100% confidence intervals 83–100%, specificity 88% CI 74–95%).

### Definitions of acquisition and colonisation

PCR results (CT values) were converted to a binary value. A CT value of 40 or more was considered negative, and CT<40 positive, for all assays apart from *E*. *coli* and *Acinetobacter*. *E*. *coli* and *Acinetobacter* assays were considered negative if > or = 35, and positive if<35 because there was evidence of PCR mastermix (*E*. *coli*) and DNA extraction buffer fluid (*Acinetobacter*) reagent contamination from negative control samples. Despite changing to a “cleaner” batch of mastermix, testing negative control samples with the *E*. *coli* assay still produced some CT values of >35, and for this reason a higher threshold was applied to accepting a positive result from these assays. Nothing could be done to mitigate the effects of the contaminated buffer fluid, but all CT values for negative controls tested with the *Acinetobacter* assay were >35, so again this threshold was adopted.

Acquisition was defined as the presence of an organism in one or more samples. Colonization was defined as two or more samples positive (non-consecutive or consecutive). A “colonisation index” was calculated by dividing total number of positive samples by the number of samples taken for each organism per patient.

### Data analysis

Anonymised data were manually checked and cleaned in Microsoft Excel (versions 2007–2010) prior to analysis. Analysis was undertaken in R (R: A language and environment for Statistical computing, Vienna, Austria). Missing data were labeled as “NA” and were excluded from analysis in R. Fisher’s exact test and univariate generalized linear modelling (GLM) were used to investigate the relationships between HAP and explanatory variables, and univariate GLMs were used to investigate HAP or lower respiratory tract infections (LTTI) with explanatory variables. In order to investigate whether colonization with individual organisms was associated with dental covariates, a multivariate generalised linear model with binomial error structure (analogous to multiple logistic regression) was used to test a dental model. Factors included in the model were age, clinical frailty score, Barthel index, Charlson comorbidity index, deprivation index, place of residence, number of teeth, presence of dentures, gender, presence of active cancer, smoking status and plaque score at admission. These factors were included as putative drivers of colonization, based on previous observational studies [16, 28, 44–47]. To our knowledge, deprivation has not been studied as a risk factor for colonization with potential respiratory pathogens but seemed a sensible additional risk factor given that it drives many of the other factors e.g. smoking, tooth number. Patients who died were included in the analysis.

## Results

**Fig 1:**
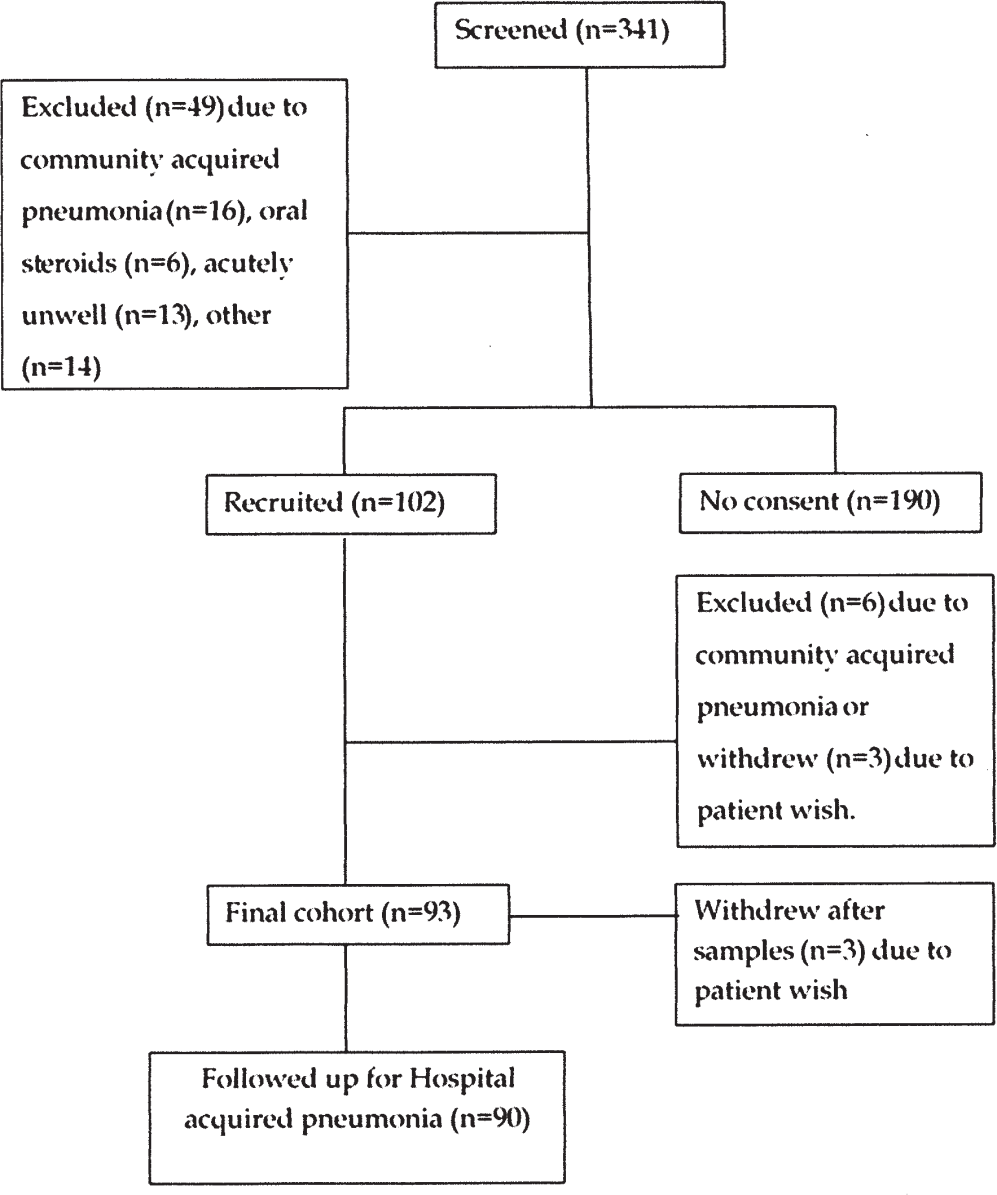
Consort diagram of screened study participants.

341 patients were screened, with a final cohort of 90 patients who were followed up for HAP (Fig 1) and 93 patients from whom oral sampling data were available. Recruitment was difficult due to timing, and there were periods where there were few potential participants. This led to under-recruiting to the study, and the recruitment period could not be extended. Reasons for non-participation of eligible patients included logistic (n = 52 e.g. no personal consultee), moribund/aggressive (n = 35), in pain/too tired (n = 20), or no reason given (n = 83). Patients admitted on Friday/Saturday were frequently not recruited, which may have led to a more “well” cohort given the increased risks of weekend hospital admission [48]. 14/90 (16%) needed a personal or professional consultee. One patient was lost to follow-up, but his GP confirmed he had received no antibiotics post-discharge and he was therefore treated as not having developed HAP/LRTI. Three patients were found to be positive for MRSA and were treated with chlorhexidine mouth rinse and medicated toothpaste as part of the hospital’s decontamination protocol. None of these patients developed HAP.

816 samples were collected from 93 patients (408 = tongue swabs, 408 = throat swabs). Samples were taken at four or five time-points from 83 participants, at two or three time-points in eight participants, and once in two participants (first sample preoperatively n = 50, post-operatively n = 43). Eleven patients missed one sample for logistic reasons.

The majority of patients (86/93) had sustained fractures to the neck of femur; others had fractured the femoral shaft (n = 2) or ankle (n = 5). Two patients did not undergo operation (stable ankle fractures). Three patients withdrew after sampling but before follow up, all of whom had sustained fractures to their neck of femur, leaving a final cohort of 90. Three patients had been decolonised for MRSA. Baseline characteristics of the study cohort are shown in Table 1. Fifty patients possessed teeth (median 16.5, range 1–50), of which 24 also wore dentures, a further 42 only wore dentures (no teeth) and one person had neither teeth nor wore dentures. Of dentate patients, the dental plaque indices at admission ranged from 0.2–3.5 with median score 1.2. Of those who wore dentures, scores ranged from 0–1.5, with a median score of 0.55 in dentate and 0.7 in edentulous persons (p = <0.001). Dental plaque but not denture plaque scores increased generally over the three examinations, but non-significantly. Combined dental/denture plaque scores had a median value of 2 (range 1–4).

**Table 1:** Baseline characteristics of study cohort, shown by patients with or without HAP (Fisher’s exact test).

*Variable*	*All patients (n = 90) Mean, Median (range)* ^1^	*HAP (n = 10) Mean, Median (range)* ^1^	*No HAP (n = 80) Mean, Median (range)* ^1^	*P value*	*Odds ratio(96% confidence interval) to 2 d.p.*
>2 samples positive, any bug	53/90	8/10	45/80	0.188	3.08 (0.56–31.53)
>2 samples positive, opportunistic orgs only^1^	17/90	6/10	11/80	0.003*	9.05 (1.82–51.27)
*S*. *aureus* col index	0.06,0 (0–1)	0.14, 0 (0–1)	0.05,0 (0–1)	0.091	
MRSA col index	0.03, 0 (0–1)	0.05,0 (0–0.37)	0.03, 0 (0–1)	0.197	
*E*. *coli* col index	0.03,0 (0–1)	0.14,0 (0–1)	0.02,0 (0–0.5)	0.036*	
*P*. *aeruginosa* col ind	0.03,0 (0–0.5)	0.05,0 (0–0.4)	0.03,0 (0–0.5)	0.125	
*S*. *pneumoniae* col ind	0.25,0 (0–1)	0.12.0 (0–0.87)	0.27,0 (0–1)	0.771	
*H*. *influenzae* col ind	0.08,0 (0–0.9)	0.05,0 (0–0.25)	0.09,0 (0–0.9)	0.330	
*Acinetobacter* sp col ind	0.01, 0 (0–0.33)	0,0 (0)	0.02,0 (0–0.33)	1	
Age	81	83.5	80.58	0.061	
Female	61/90	5/10	56/80	0.281	0.43 (0.09–2.07)
Clinical Frailty Scale	4.29	5	4.2	0.570	
Charlson index	5	7.4	5.1	0.067	
HABAM (mobility score)	52	45.7, 50.5 (18–61)	52.4	0.194	
Barthel score	18.17, 20 (4–20)	16.1, 19 (4–20)	18.43, 20 (4–20)	0.143	
Number of teeth	8.5, 5 (0–28)	10, 7 (0–27)	8.3, 3.5 (0–28)	0.353	
Denture wearing	64/90 (71%)	8/10	56/80	0.718	1.71 (0.31–17.66)
Home dwelling	75/90 (83%)	6/10	69/80	0.058	0.24 (0.05–1.37)
PQS day 1 n = 89	2.4, 2 (1–4)	1.9, 3 (1–4)	2.5, 2 (1–4)	0.265	
PQS day 7 n = 78	2.7, 3 (1–4)	3, 3.5 (1–4)	2.6, 3 (1–4)	0.403	
PQS day 14 n = 61	2.5, 3 (1–4)	3, 3 (1–4)	2.4, 2 (1–4)	0.451	
Deprivation score	26.2, 20.5 (2.3–71.7)	18.67, 14.0 (3.7–49.5)	27.1, 22.4 (2.3–71.7)	0.058	
Number of drugs (admission)	6	5.9	6.3	0.758	
Proton pump inhibitor	26/90	2/10	24/80	0.718	0.59 (0.06–3.25)
Angiotensin converting enzyme inhibitor	20/90	1/10	19/80	0.448	0.36 (0.01–2.90)
Sedating drugs^a^	32/90	6/10	26/80	0.157	3.07 (0.66–16.15)
Inhaled steroid	14/90	0/10	14/80	0.351	0.00 (0.00–2.41)
Oral steroid (<7.5mg)	6/90	1/10	5/90	0.517	1.66 (0.03 17.49)
Statin	48/90	7/10	41/80	0.327	2.20 (0.46–14.12)
Cefuroxime perioperatively	20/87	3/10	17/77	0.690	0.66 (0.13–4.41)
Teicoplanin perioperatively	67/87	7/10	60/77	NA	
Number of comorbidities n = 89	6	6.4	6.4	0.913	
Number of complications	10, 8 (0–50)	21.6, 18.5 (5–50)	8.4, 7 (0–36)	0.001*	
COPD	17/90 (19%)	1/10	16/80	0.680	0.45 (0.01–3.67)
Any respiratory comorbidity	21/90 (23%)	3/10	18/80	0.693	1.47 (0.22–7.30)
Cerebrovascular disease	16/90 (18%)	1/10	15/80	0.684	0.48 (0.01–4.00)
Cardiovascular disease	30/90 (33%)	3/10	27/80	1	0.84 (0.13–4.06)
Diabetes mellitus	9/90 (10%)	2/10	7/80	0.261	2.57 (0.22–17.15)
Dementia	6/90 (7%)	2/10	4/80	0.131	4.62 (0.36–38.76)
Active cancer	4/90 (4%)	4/10	3/80	0.002*	15.97 (2.18–136.36)
Antibiotics pre admission	15/90 (17%)	3/10	12/80	0.361	2.40 (0.35–12.51)
Current smoking	14/90 (16%)	2/10	12/80	0.651	1.41 (0.13–8.40)
Current or ex smoking	60/90 (67%)	8/10	52/80	0.486	2.4 (0.39–22.0)
Operation length (mins) n = 87	88.90	91.50	88.56	0.175	
Witnessed aspiration episode	4/90 (4%)	3/10	1/80	0.004*	30.88 (2.18–1773.08)
Length of stay (days)	38, 26 (4–265)	54.9, 46 (13–140)	36.31, 24.50 (4–265)	0.010*	
Death (all causes)	15/90 (17%)	8/10	7/80	<0.001*	38.18 (6.14–434.01)
Death (excluding active cancer) n = 83	11/83 (12%)	4/10	7/73	0.001*	17.23 (2.44–203.26)

HAP = hospital acquired pneumonia, HABAM = Hierarchical Assessment of Balance and Mobility, PQS = Plaque quartile score, COPD = Combined obstructive pulmonary disease, d.p. = decimal places

*statistically significant p<0.01

^a^includes benzodiazepines, selective serotonin reuptake inhibitors, tricyclic antidepressants, opiates, gabapentin or anti-epileptic drugs.

^1^Where mean and median were similar, only the mean is shown. Where there was large disparity between these values, mean, median and range are given.

### Microbiological sampling

Of 93 patients, 23 patients had negative swabs and a further 26 had transient acquisition of organisms. The other 44 had single (n = 22) or mixed pathogen carriage/acquisition (n = 22). Of 51 colonisation events (one patient could have >1), colonisation with *S*. *pneumoniae* was commonest (n = 27), followed by *H*. *influenzae* (n = 8), *S*. *aureus (n = 6)*, *P*. *aeruginosa* (n = 4), MRSA (n = 3) and *E*. *coli*/*Acinetobacter* spp (both n = 2). Certain combinations of pathogens were seen more frequently. Disregarding the persistence of the organisms, the commonest combinations of organisms were *S*. *pneumoniae* and *H*. *influenzae* (n = 11), *S*. *aureus* and MRSA (n = 10), *E*. *coli* and *H*. *influenzae* (n = 5), and *S*. *pneumoniae* and *P*. *aeruginosa* (n = 5). Colonisation indices were highest in *S*. *pneumoniae*, indicating most persistent carriage.

**Fig 2:**
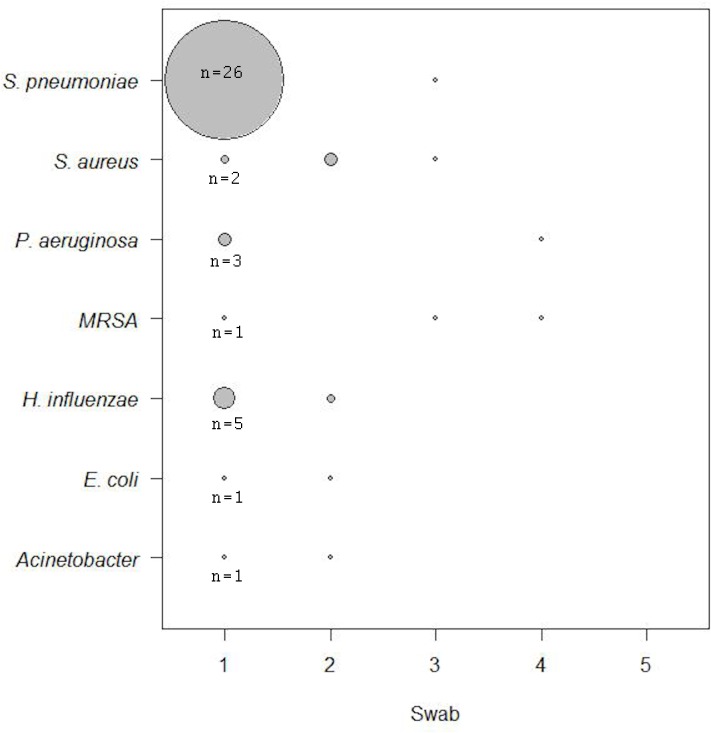
Diagrammatic representation of the time-point during admission that each organism was first detected (when an organism was detected twice or more, i.e. colonisation). Note that these data are not cumulative, because some organisms might have been detected on the first sample, not on the second and then been detected again on the third sample. The shaded area of each circle represents the number of patients in whom colonisation with that organism was detected at that time-point. The vast majority of these organisms were first acquired on sample 1 or 2 (i.e. during the initial 3 days in hospital), and not later in the hospital stay.

Colonising organisms (i.e. detected twice or more) were first detectable within 72 hours of admission in 90% of cases (Fig 2).

### Hospital Acquired Pneumonia

Ten of 90 patients were defined as having HAP (clinician initiated antibiotics), of whom seven fulfilled ATS/BSAC criteria, one who was too ill for a chest radiograph and died (death certificate recorded 1a as being pneumonia) and two others who had new infiltrates on chest radiograph but had only one of the minor criteria. A further eight patients developed LRTI. Two anonymised datasets are included (S1 Dataset: Anonymised patient data with PCR results (CT values) and S2 Dataset: Anonymised patient results with PCR results expressed as colonisation indices) comprising PCR results from these 90 patients, along with other covariates studied. A third file (S3 Dataset) contains the codes of categorical data within the dataset.

**Fig 3:**
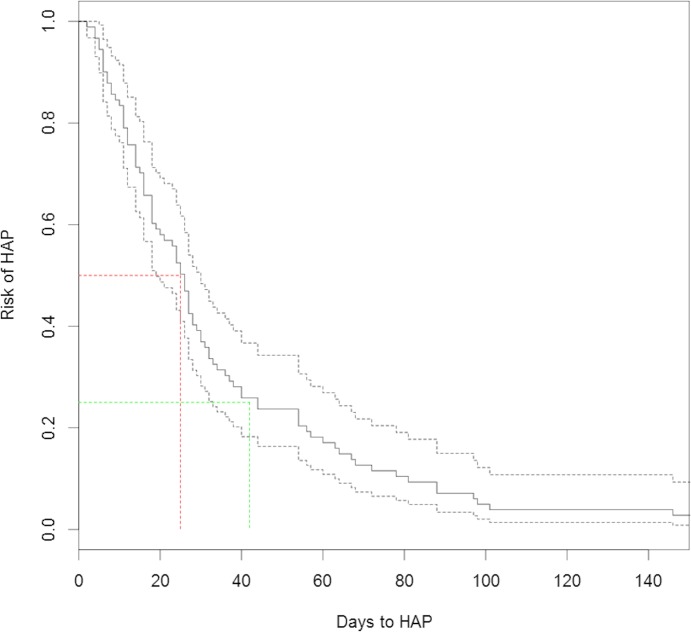
Risk of HAP by number of days in hospital. The risk of HAP declined with number of days in hospital, with the highest risk being in the first six weeks (green dashed lines). Around 50% of cases occurred within the first 25 days (red dashed lines).

Two patients grew *S*. *pneumoniae* from sputum and a third grew *H*. *influenza*e (all three carried *S*. *pneumoniae* on oral study samples). Of the ten patients defined as having HAP, eight (80%) died, six in hospital and two after discharge. Total number of days in hospital for the 90 patients was 3454, and each patient was also followed up for 90 days post discharge (8100 days), giving a total of 11,554 days studied, and thus risk of first episode of HAP per day was 0.0009. Around 50% of cases arose within the first 25 days in hospital, and 75% occured within the first six weeks of admission (Fig 3). Having HAP was associated with a significantly increased length of stay (Fisher’s exact test, p = 0.01), with a mean excess number of days in hospital per person of 30 days (range -11.5–115).

HAP was not associated with being dentate, tooth number, having dentures or combined denture/dental plaque score at any time point during admission (Tables 1 and 2). HAP was not significantly associated with 2 or more positive samples (colonisation) of a combined group of all organism tested. However it was noted that the estimates for *S*. *pneumoniae* and *H*. *influenzae* were negative, suggesting a trend towards a protective effect. Thus we combined those organisms with models which had positive estimates into one group (‘opportunistic organisms’), which comprised *S*. *aureus*, MRSA, *P*. *aeruginosa* and *E*. *coli*. We did not include *Acinetobacter* in this group because the model produced by relating HAP with *Acinetobacter* spp was so poor (probably because only three patients were colonised with *Acinetobacter* spp—one with three samples positive and the other two with two samples positive). When we removed *S*. *pneumoniae*, *H*. *influenzae* and *Acinetobacter* spp. from the group, HAP was significantly associated with prior colonisation by the opportunistic organisms group by univariate GLM (p = 0.002**, odds ratio 9.41 95% confidence intervals 2.28–38.78). The adjusted r-squared value for this model was 0.203, suggesting that the presence of these organisms in the oropharynx explained 20% of the cases of HAP. The crude incidence of HAP in unexposed patients was 3/73 (4.1%), while in exposed patients crude incidence of HAP was 6/17 (35.3%), a difference of 31.2%. The relative risk of developing HAP if colonised with these organisms was 6.44 (95% CI 2.04–20.34, p = 0.002).

**Table 2:** Relating HAP and patient factors using univariate generalised linear modelling.

Variable	Estimate	Standard error	Z value	Null deviance		Residual deviance	P value	Odds ratio (95% confidence intervals)to 2 d.p.
>2 samples positive, any bug	1.135	0.822	1.381	62.790 on 89	60.541 on 88		0.167	3.11 (0.62–15.58)
>2 samples positive, opportunistic orgs only^1^	2.242	0.723	3.103	62.790 on 89	53.084 on 88		0.002 **	9.41 (2.28–38.78)
*S*. *aureus* col index	2.136	1.447	1.476	62.79 on 89	60.90 on 88		0.14	8.46 (0.50–144.33)
MRSA col index	1.146	1.980	0.579	62.790 on 89	62.502 on 88		0.563	3.14 (0.06–152.53)
*E*. *coli* col index	4.456	2.459	1.812	62.790 on 89	58.046 on 88		0.07.	86.17 (0.70–10680.08)
*P*. *aeruginosa* col ind	2.496	3.231	0.773	62.790 on 89	62.267 on 88		0.44	12.14 (0.02–6827.16)
*S*. *pneumoniae* col ind	-1.390	1.224	-1.136	62.790 on 89	61.141 on 88		0.256	0.25 (0.02–2.74)
*H*. *influenzae* col ind	-1.449	2.471	-0.586	62.790 on 89	62.367 on 88		0.558	0.23 (0.00–29.79)
*Acinetobacter* sp col ind	-153.807	15919.098	-0.01	62.79 on 89	60.81 on 88		0.992	NA
Age	0.058	0.049	1.175	62.790 on 89	61.322 on 88		0.240	1.06 (0.96–1.17)
Clinical frailty scale	0.334	0.221	1.510	62.790 on 89	60.432 on 88		0.131	1.40 (0.91–2.15)
Decreased mobility	-0.048	0.027	-1.767	62.790 on 89	59.817 on 88		0.077	0.95 (0.90–1.01)
Barthel index	-0.121	0.069	-1.76	62.790 on 89	60.054 on 88		0.078.	0.89 (0.77–1.01)
Charlson index	0.382	0.137	2.788	62.790 on 89	54.803 on 88		0.005 **	1.46 (1.12–1.92)
Number of teeth	0.017	0.033	0.516	62.790 on 89	62.528 on 88		0.606	1.02 (0.95–1.09)
Day 1 PQS	-0.492	0.323	-1.522	62.553 on 88	59.995 on 87		0.128	0.61 (0.32–1.15)
Day 7 PQS (n = 78)	0.3261	0.358	0.912	51.586 on 77	50.704 on 76		0.362	1.39 (0.69–2.79)
IMD	-0.026	0.021	-1.258	62.553 on 88	60.718 on 87		0.208	0.97 (0.94–1.01)
Female	-0.847	0.678	-1.250	62.790 on 89	61.255 on 88		0.211	0.43 (0.11–1.62)
HABAM (mobility)	-0.048	0.027	-1.767	62.790 on 89	59.817 on 88		0.077.	0.95 (0.90–1.01)
Denture wearing	0.539	0.827	0.651	62.790 on 89	62.328 on 88		0.515	1.71 (0.34–8.68)
Not home dwelling	1.431	0.723	1.980	62.790 on 89	59.213 on 88		0.048 *	4.18 (1.01–17.23)
Number of drugs	-0.027	0.085	-0.318	62.790 on 89	62.686 on 88		0.750	0.97 (0.82–1.15)
PPI	-0.539	-0.539	-0.651	62.790 on 89	62.328 on 88		0.515	0.58 (1.68–0.20)
ACE-I	-1.031	1.086	-0.949	62.790 on 89	61.653 on 88		0.343	0.36 (0.04–3.00)
Sedating drug	1.136	0.688	1.651	62.790 on 89	59.996 on 88		0.099.	3.12 (0.81–12.00)
Inhaled steroids	-16.679	1743.249	-0.010	62.790 on 89	59.185 on 88		0.992	NA
Oral steroids <7.5mg	0.511	1.151	0.444	62.790 on 89	62.611 on 88		0.657	1.67 (0.17–15.90)
Statin	0.797	0.725	1.099	62.790 on 89	61.494 on 88		0.272	2.22 (0.54–9.20)
Number of comorbidities	-0.004	0.113	-0.037	62.790 on 89	62.788 on 88		0.97	1.00 (0.80–1.24)
COPD	-0.811	1.091	-0.744	62.790 on 89	62.126 on 88		0.457	0.44 (0.05–3.77)
Any resp	0.390	0.740	0.526	62.790 on 89	62.524 on 88		0.599	1.48 (0.35–6.30)
Cerebrovascular disease	-0.731	1.092	-0.669	62.790 on 89	62.263 on 88		0.503	0.48 (0.06–4.10)
Diabetes mellitus	0.958	0.884	1.084	62.790 on 89	61.757 on 88		0.278	2.61 (0.46–14.75)
Dementia	1.558	0.942	1.653	62.790 on 89	60.473 on 88		0.098.	4.75 (0.75–30.12)
Cardiovascular disease	-0.173	0.729	-0.237	62.790 on 89	62.733 on 88		0.813	0.84 (0.20–3.51)
Active cancer	2.840	0.874	3.251	62.790 on 89	52.641 on 88		0.001 **	17.11 (3.09–94.80)
Antibiotics pre admit	0.887	0.758	1.171	62.790 on 89	61.539 on 88		0.242	2.43 (0.55–10.73)
Current smoking	-0.348	0.850	-0.410	62.790 on 89	62.631 on 88		0.682	0.71 (0.13–3.74)
Ex or current smoker	-0.767	0.825	0.8246	62.790 on 89	61.817 on 88		0.352	0.46 (0.09–2.34)
Length of stay (days)(log)	0.825	0.413	1.999	62.790 on 89	58.503 on 88		0.046 *	2.28 (1.02–5.12)
Witnessed aspiration	3.522	1.220	2.887	62.790 on 89	53.031 on 88		0.004 **	33.86 (3.10–370.07)

Abbreviations: HAP = hospital acquired pneumonia, d.p. = decimal places, PQS = plaque quartile score, IMD = Index of multiple deprivation score, HABAM = Hierarchical Assessment of Balance and Mobility, PPI = proton pump inhibitor, ACE-I = Angiotensin converting enzyme inhibitor, Cef vs teic = Whether the patient received Cefuroxime or Teicoplanin perioperatively, COPD = Combined obstructive pulmonary disease, Any resp = Any respiratory comorbidity, log = logarithim

When testing for associations between HAP or HAP/LRTI and single organisms, only *E*. *coli* (HAP, p = 0.036*) or *S*. *aureus* (HAP/LRTI, p = 0.028, OR 25.95, 95%CI 1.43–471.92) were significantly associated (Tables 1 and 3), though HAP was not significantly associated with *E*. *coli* when using a GLM (Table 2, p = 0.07).

**Table 3:** Relating HAP/LRTI and patient factors using univariate generalised linear modelling.

Variable	Estimate	Standard error	Z value	Null deviance		Residual deviance	P value	Odds ratio (95% confidence intervals)to 2 d.p.
>2 samples positive, any bug	0.732	0.577	1.269	90.072 on 89	88.358 on 88		0.205	2.08 (0.67–6.45)
>2 samples positive, opportunistic orgs only^1^	1.723	0.593	2.904	90.072 on 89	81.829 on 88		0.004 **	5.60 (1.75–17.92)
*S*. *aureus* col index	3.256	1.480	2.201	90.072 on 89	84.580 on 88		0.028 *	25.95 (1.43–471.92)
MRSA col index	3.484	2.086	1.670	90.072 on 89	86.456 on 88		0.095.	32.59 (0.54–1944.49)
*E*. *coli* col index	2.802	2.008	1.395	90.072 on 89	87.789 on 88		0.163	16.48 (0.32–843.60)
*P*. *aeruginosa* col ind	0.301	3.126	0.096	90.072 on 89	90.063 on 88		0.923	1.35 (0.00–619.09)
*S*. *pneumoniae* col ind	-1.303	0.894	-1.457	90.072 on 89	87.503 on 88		0.145	0.27 (0.05–1.57)
*H*. *influenzae* col ind	0.619	1.345	0.460	90.072 on 89	89.870 on 88		0.645	1.86 (0.13–25.94)
*Acinetobacter* sp col ind	-6.336	8.645	-0.733	90.072 on 89	89.295 on 88		0.464	NA
Age	0.062	0.039	1.577	90.072 on 89	87.378 on 88		0.115	1.06 (0.99–1.15)
Clinical frailty scale	0.527	0.196	2.682	90.072 on 89	81.648 on 88		0.007 **	1.69 (1.15–2.49)
Barthel index	-0.078	0.062	-1.253	90.072 on 89	88.605 on 88		0.210	0.93 (0.82–1.04)
Charlson index	0.389	0.119	3.267	90.072 on 89	78.302 on 88		0.001 **	1.48 (1.17–1.86)
Number of teeth	0.017	0.026	0.650	90.072 on 89	89.656 on 88		0.516	1.02 (0.97–1.07)
Day 1 PQS	0.327	0.236	1.385	89.623 on 88	87.645 on 87		0.166	1.39 (0.87–2.20)
Day 7 PQS	0.367	0.268	1.368	79.159 on 77	77.171 on 76		0.171	1.44 (0.85–2.44)
IMD	-0.004	0.014	-0.322	89.623 on 88	89.518 on 87		0.748	1.00 (0.97–1.02)
Female	-0.956	0.540	-1.770	90.072 on 89	86.971 on 88		0.077.	0.38 (0.13–1.11)
HABAM (mobility)	-0.047	0.023	-2.064	90.072 on 89	85.788 on 88		0.039 *	0.95 (0.91–1.00)
Denture wearing	0.432	0.622	0.694	90.072 on 89	89.566 on 86		0.488	1.54 (0.46–5.21
Home dwelling	0.869	0.627	1.386	90.072 on 89	88.265 on 88		0.166	2.38 (0.70–8.15)
Number of drugs	0.062	0.062	1.007	90.072 on 89	89.075 on 88		0.314	1.064 (0.94–1.20)
PPI	-0.068	0.587	-0.116	90.072 on 89	90.059 on 88		0.907	0.93 (0.30–2.95)
ACE-I	-0.435	0.691	-0.63	90.072 on 89	89.650 on 88		0.529	0.65 (0.17–2.51)
Sedating drug	1.044	0.539	1.937	90.072 on 89	86.288 on 88		0.053.	2.84 (0.99–8.17)
Inhaled steroids	-0.470	0.814	-0.577	90.072 on 89	89.711 on 88		0.564	0.63 (0.13–3.08)
Oral steroids <7.5mg	0.754	0.910	0.829	90.072 on 89	89.440 on 88		0.407	2.13 (0.36–12.63)
Statin	0.112	0.530	0.211	90.072 on 89	90.028 on 88		0.833	1.12 (0.40–3.16)
Number of comorbidities	0.129	0.086	1.494	90.072 on 89	87.841 on 88		0.135	1.14 (0.96–1.35)
COPD	0.260	0.644	0.403	90.072 on 89	89.914 on 88		0.687	1.30 (0.37–4.58)
Any resp	0.642	0.578	1.110	90.072 on 89	88.888 on 88		0.267	1.90 (0.61–5.90)
Cerebrovascular disease	-0.097	0.703	-0.138	90.072 on 89	90.053 on 88		0.89	0.91 (0.23–3.60)
Diabetes mellitus	0.789	0.763	1.034	90.072 on 89	89.082 on 88		0.301	2.20 (0.49–9.81)
Dementia	1.526	0.865	1.765	90.072 on 89	87.147 on 88		0.078	4.60 (0.84–25.06)
Cardiovascular disease	0.598	0.539	1.109	90.072 on 89	88.862 on 88		0.267	1.82 (0.63–5.23)
Active cancer	2.600	0.890	2.923	90.072 on 89	80.425 on 88		0.003**	13.46 (2.35–76.95)
Antibiotics pre admit	1.253	0.614	2.040	90.072 on 89	86.141 on 88		0.041 *	3.50 (1.05–11.66)
Current smoking	-0.104	0.712	-0.145	90.072 on 89	90.052 on 88		0.884	0.90 (0.22–3.64)
Ex or current smoker	-0.682	0.618	-1.104	90.072 on 89	88.753 on 88		0.269	0.51 (0.15–1.70)
Length of stay (days)(log)	0.925	0.346	2.678	90.072 on 89	81.901 on 88		0.007 **	2.52 (1.28–4.97)
Witnessed aspiration	2.653	1.189	2.231	90.072 on 89	84.104 on 88		0.026 *	14.20 (1.38–146.06)

Abbreviations: HAP = hospital acquired pneumonia, LRTI = lower respiratory tract infection, d.p. = decimal places, PQS = plaque quartile score, IMD = Index of multiple deprivation score, HABAM = Hierarchical Assessment of Balance and Mobility, PPI = proton pump inhibitor, ACE-I = Angiotensin converting enzyme inhibitor, Cef vs teic = Whether the patient received Cefuroxime or Teicoplanin perioperatively, COPD = Combined obstructive pulmonary disease, Any resp = Any respiratory comorbidity

^1^: includes *S*. *aureus*, MRSA, *P*. *aeruginosa*, *E*. *coli*

We also tested whether HAP was associated with samples positive for any of these opportunistic organisms at each time point. HAP was significantly associated with opportunistic organisms being detected on oral samples at day 5 or 14 (Table 4).

**Table 4:** Association between HAP and presence of opportunistic organisms by day sampled.

Day	Estimate	Standard error	Z value	Null deviance	Residual deviance		P value	Odds ratio (95% confidence intervals)to 2 d.p.
1	0.758	0.470	1.612	154.55 on 199		152.14 on 198	0.107	2.13 (0.85–5.36)
3	0.460	0.614	0.749	107.42 on 176		106.90 on 175	0.454	1.58 (0.48–5.28)
5	1.479	0.476	3.105	139.61 on 173		130.63 on 172	0.002 **	4.39 (1.73–11.16)
7	1.099	0.596	1.842	99.953 on 161		96.904 on 160	0.065.	3.00 (0.93–9.65)
14	1.900	0.522	3.644	117.70 on 137		104.95 on 136	<0.001 ***	6.69 (2.40–18.60)

HAP was associated with higher Charlson index (Table 2, p = 0.005, OR 1.46, 95%CI 2.28–38.78), being admitted from hospital/institution (0.048, OR 4.18, 95% CI 1.01–17.23), having active cancer (p = 0.001, OR 17.11, 95% CI 3.09–94.80), or having a witnessed aspiration episode (p = 0.004, OR 33.86, 95% CI 3.10–370.07). It should be remembered that aspiration episodes were not collected systematically from all patients and this result may therefore be biased. Having metastatic cancer adds six points to the Charlson index, while other comorbidities (other than HIV) only add one to two points, which may explain the collinearity seen between active cancer and increased Charlson index.

HAP/LRTI was also associated with increased Charlson index (p = 0.001, OR 1.48, 95%CI 1.17–1.86), having active cancer (p = 0.003, OR 13.46, 95%CI 2.35–76.95), witnessed aspiration episodes (p = 0.026, OR 14.20, 95% CI 1.38–146.06), and >2 samples positive for opportunistic organisms (p = 0.004, OR 5.60, 95% CI 1.75–17.92) (Table 3). Additionally, HAP/LRTI was associated with increased clinical frailty score (p = 0.007, OR 1.69, 95% CI 1.15–2.49), having had antibiotics before hospital admission (p = 0.041, OR 3.50, 95% CI 1.05–11.66), and worse mobility score (p = 0.039, OR 0.95, 95% CI 0.91–1.00). We did not undertake multivariate regression because of the small number of cases in this study.

We then created a correlation matrix to look for collinearity between the significant variables described above (Table 5). Significant collinearity was defined as an estimate greater than 0.2 (or less than-0.2), and was seen between worse mobility/increased frailty and being admitted from an institution/hospital, between increased frailty and worse mobility, increased Charlson index and active cancer, and between active cancer and witnessed aspiration episodes. Colonisation with opportunistic organisms was most strongly collinear with having active cancer and witnessed aspiration episodes.

**Table 5:** Colinearity between covaiates which were significantly associated with HAP.

	*Admitted from hospital/ institution*	*HABAM*	*Charlson Index*	*Abx pre admission*	*Witnesed aspiration*	*Active cancer*	*>2 samples positive with opportunistic orgs.*	*Clinical frailty score*
Admitted from hosp./inst.	1	Pearson	Pearson	Polyserial	Polyserial	Polyserial	Pearson	Pearson
HABAM	**-0.516**	1	Pearson	Polyserial	Polyserial	Polyserial	Pearson	Pearson
Charlson Index	0.144	**-0.368**	1	Polyserial	Polyserial	Polyserial	Pearson	Pearson
Abx pre admission	0.040	-0.123	0.136	1	Polychoric	Polychoric	Polyserial	Polyserial
Witnessed aspiration	**0.273**	**-0.321**	**0.440**	**-0.646**	1	Polychoric	Polyserial	Polyserial
Active cancer	-0.031	0.046	**0.522**	-0.099	**0.617**	1	Polyserial	Polyserial
>2 samples pos. opp. orgs.	-0.059	0.004	0.160	0.171	**0.225**	**0.269**	1	Pearson
Clinical frailty score	**0.481**	**-0.759**	**0.362**	0.163	**0.394**	**0.246**	-0.018	1

Abbreviations: HABAM = Hierarchical Assessment of Balance and Mobility, ABX = antibiotics, orgs. = organisms. Variables which were co-linear (correlation estimate >0.20) are highlighted in boldface. The higher the number, the stronger the correlation. Negative correlation estimates mean the variable on the ‘y’ axis was associated with a lower score of the variable in the ‘x’ axis (e.g. higher Charlson index was associated with a lower or worse HABAM/mobility score). Where covariates are binomial (e.g. Admitted from hospital/institution, a negative correlation estimate refers to that event NOT having happened (e.g. higher HABAM/mobility score was associated with not having been admitted from hospital/institution, or in other words, having been admitted from home.). Covariates in the ‘y’ axis have been abbreviated to make viewing the table easier.

Given that previous studies found respiratory tract infection or aspiration pneumonia was associated with poor dentition, while we found no associations, we investigated whether colonisation with any organism was associated with dental factors (S2 Table) using the dental model (described above in Methods). Being colonised with *E*. *coli* was significantly commoner in those without teeth or dentures, and increased *S*. *aureus* colonisation was seen in those with higher admission plaque scores. In keeping with the notion of *S*. *pneumoniae* being protective against HAP, colonisation with *S*. *pneumoniae* was associated with having more teeth and being less frail. Smoking was a common risk factor for all organisms studied other than *H*. *influenzae*. Interestingly *S*. *pneumoniae* was also associated with being less deprived, while *H*. *influenzae* was associated with being more deprived.

## Discussion

In this study, HAP was not associated with tooth number or prior heavy dental/denture plaque, but was significantly associated with two or more samples positive with either *S*. *aureus*, MRSA, *E*. *coli* or *P*. *aeruginosa* at any time point, and specifically at days 5 and 14 after admission. One previous study reported finding a significant association between aspiration pneumonia and *S*. *aureus* in saliva [29], and these findings are similar to those from patients with VAP [49, 50], but there are no other studies with which to compare these findings in non-ventilated HAP, to our knowledge. Both studies of oropharyngeal colonization in VAP patients noted that different outcomes were associated with colonization by two groups of organisms- a *S*. *pneumoniae*/ *H*. *influenzae* group and an Enterobactericeae/ *P*. *aeruginosa* group [49, 50] (*S*. *aureus* was placed in the first [50], and the second group [49]). HAP resulted in a mean of 30 excess days in hospital per patient and 50% of cases occurred in the first 25 days of admission. In addition, patients with higher Charlson indices or active cancer were at increased risk of HAP.

While this was a small study, it combined dental covariates with microbial data detected by real-time PCR, using purpose-designed assays for clinically relevant organisms, and repeated sampling to improve detection of colonization over time. While oropharyngeal colonization by potentially pathogenic organisms has been previously described in older hospitalized persons [12, 33, 34, 46, 51, 52], molecular methods have not been previously used. The study added useful information regarding incidence of HAP in persons colonised and uncolonised by opportunistic organisms, which may inform power calculations for future intervention trials. The study added data concerning the timing of first colonization event, important information about the significance of the presence of opportunistic organisms later in hospital admission, baseline dental data from hospital patients and also data regarding excess length of stay. Despite the size, the study detected differences between incidence of HAP in exposed and unexposed participants of 31.2%, and the incidence of HAP in unexposed persons was 4.1%, which was lower than that used in the power calculation. The organisms implicated in this study might be piloted as endpoints to determine the effectiveness of oral hygiene interventions in intervention trials.

Colonisation with opportunistic organisms only explained 20% of the variance, suggesting other risk factors are of importance- i.e. that the presence of these organisms alone does not necessarily lead to HAP. Other significant risk factors for HAP in this study suggest the hypothesis that significant multimorbidity (increased Charlson index) and/or active cancer drive both colonization with opportunistic organisms and also tendency to aspiration, possibly via frailty. Patients who aspirate may have impaired swallow reflexes that decrease mechanical clearance in the mouth, both increasing colonization with opportunistic organisms [53] and the likelihood that these organisms will be delivered to the bronchial tree. Structural equation modelling, which incorporates a temporal element, might clarify these interactions more realistically, using conceptual models as described by Dickson et al. [54]. Interventions ought to address as many risk factors as possible to prevent HAP, by promoting an aerodigestive tract which minimizes delivery of organisms to the bronchial tree, impede bacterial overgrowth, and optimize the habitat for core oral microbiota. Such interventions might include improving swallow (ACE-inhibitors or oral hygiene), reducing bacterial bioburden (oral hygiene), avoiding decreased conscious level (avoiding opiates, sedating drugs, general anaesthetics), use of probiotics, optimising nutritional intake, promoting upright positioning and mobility, and avoiding indwelling plastics which rapidly become colonized, such as nasogastric tubes, where possible.

Future HAP studies should include a bedside swallow assessment in order to systematically assess aspiration risk. Larger studies are needed to trial possible interventions and in order to undertake multivariate modelling, perhaps aiming to recruit 100 cases of HAP. Oral colonisation with respiratory pathogens began within 72 hours of admission in the majority of cases, implicating either events early in admission or even in the community, rather than the hospital environment, as the source of opportunistic organisms. Thus interventions to manipulate the oral microbiota and subsequent risk of pneumonia might begin within this timeframe, and pre-operatively where appropriate. In addition, given that potentially pathogenic organisms were found on the tongue and the throat, oral hygiene interventions ought to be directed to, but not necessarily limited to, the tongue and throat.

The results from this study are not directly generalizable to medical patients because study patients experienced fracture, pain, anaesthesia and operation. However the organisms implicated in this study are also seen more frequently with increasing severity of COPD [51], and *S*. *aureus* has also previously been found to be a risk factor for aspiration pneumonia in medical patients[12]. It is unfortunate that we were not able to satisfactorily develop and validate a PCR assay to look for *Klebsiella pneumoniae* or other Enterobactericeae because several studies have noted the increased prevalence of these organisms in frail, institutionalized patients, and in those who are increasingly unwell [33, 34, 46, 52], and it seems possible that these organisms might have represented an additional risk factor for HAP. It should be noted that some patients received broad spectrum antibiotics, and other narrower spectrum antibiotics, but that these, and the use of chlorhexidine for eradication of MRSA may have reduced detection of organisms sought in the study.

Plaque scores were moderate for the group (mean scores of >2 are thought of as high). In this small study, HAP was not associated with the presence of dental/denture plaque which was consistent with results from a study of ventilated patients [16]. This may be a real finding, or may be that the study was underpowered to detect a difference. Another explanation might be that plaque needs to be colonized with particular organisms in order to promote HAP, as described in a study of nosocomial infection in ventilated patients [16], and indeed increased colonization with *S*. *aureus* was associated with higher plaque scores.

These findings need to be interpreted in light of the relatively small sample size, which might underestimate the significance of smaller effects. The study was adequately powered (80% power, 5% significance level) to distinguish between variables where the odds ratio was just under 4, but insufficiently powered to determine smaller differences than this [32]. Despite this, significant findings were obtained, and given that there is very little data in this area, the results from this study will help to inform future studies. The sample was biased towards well patients, which may have led to underestimation of both the exposures and the main outcome variables.

Pneumonia is difficult to diagnose reliably, and incidence varies with criteria used [55]. Best diagnostic practice should include bronchoalveloar lavage [55], which was not clinically appropriate in this group of patients. Therefore it is likely that the incidence of HAP found in this study is overestimated. Well-conducted randomised-controlled trials of interventions with pragmatic clinical endpoints are probably the next step given that this group of frail patients is at high risk of HAP.

This study only sampled patients for the first 14 days after admission, and oral bacterial communities may have changed after this time, or between samples, affecting the risk of HAP at a later point in the patient’s admission. The combination of tongue/throat samples was the minimally inclusive combination achievable in those with cognitive impairment; oral rinses, the gold standard were not possible in this group [24]. It should be noted that the colonization index data were zero inflated and there is a possibility of over-predicting the significance of patient factors where colonization index was larger than zero. Nasopharyngeal sampling was not undertaken in this study, partly because there was more previous research concerning target organisms in the oropharynx in this patient group, and partly because of the additional resources which would have been needed. However we acknowledge that this approach may have decreased the yield of *S*. *pneumoniae* and *H*. *influenzae* in particular [56]. *S*. *pneumoniae* occurred more commonly in the fitter patients in this study, possibly due to greater interaction with other persons in the community or family members.

Future studies investigating HAP in non-ventilated hospital patients ought to unite oral microbiological parameters using next-generation sequencing and functional metagenomics techniques with swallowing assessment, and possibly “environmental” measures of the aerodigestive tract, to better understand how these risk factors interact with each other and patient multimorbidity. Intervention trials to prevent HAP need to determine the optimal, most cost-effective methods to prevent HAP, which might include oral hygiene, swallow assessment and management and possibly medication management. In addition, methods of implementing and delivering better oral hygiene at ward-level in a resource-scarce health system need to be investigated, in order to minimise use of antibiotics and length of hospital stay associated with HAP.

## Supporting Information

S1 Dataset Anonymised patient data with PCR results (CT values). (CSV)

S2 Dataset Anonymised patient data with PCR results expressed as colonisation indices. (CSV)

S3 Dataset Codes. (DOCX)

S1 Table Primer and probe sequences for assays used in this study. (DOCX)

S2 Table Multivariate generalised linear models relating colonisation index with patient factors. (DOCX)

## References

[1] Health Protection Agency. English National Point Prevalence Survey on Healthcare-associated Infections and Antimicrobial Use, 2011: Preliminary data Health protection Agency: London 2012.

[2] Roche JJW, Wenn RT, Sahota O, Moran CG. Effect of comorbidities and postoperative complications on mortality after hip fracture in elderly people: prospective observational cohort study. BMJ. 2005;331(7529):1374 16299013 10.1136/bmj.38643.663843.55 PMC1309645

[3] Khasraghi FA, Lee EJ, Christmas C, Wenz JF. The economic impact of medical complications in geriatric patients with hip fracture. Orthopedics. 2003;26(1):49–53; discussion 12555834 10.3928/0147-7447-20030101-14

[4] Garcia-Alvarez F, Al-Ghanem R, Garcia-Alvarez I, Lopez-Baisson A, Bernal M. Risk factors for postoperative infections in patients with hip fracture treated by means of Thompson arthroplasty. Archives of Gerontology & Geriatrics. 2010;50(1):51–5. 19233490 10.1016/j.archger.2009.01.009

[5] Rothan-Tondeur M, Meaume S, Girard L, Weill-Engerer S, Lancien E, Abdelmalak S, et al Risk factors for nosocomial pneumonia in a geriatric hospital: a control-case one-center study. Journal of the American Geriatrics Society. 2003;51(7):997–1001. 12834521 10.1046/j.1365-2389.2003.51314.x

[6] Vergis EN, Brennen C, Wagener M, Muder RR. Pneumonia in long-term care: a prospective case-control study of risk factors and impact on survival. Archives of Internal Medicine. 2001;161(19):2378–81. 11606155 10.1001/archinte.161.19.2378

[7] Celis R, Torres A, Gatell JM, Almela M, Rodriguez-Roisin R, Agusti-Vidal A, et al Nosocomial pneumonia. A multivariate analysis of risk and prognosis. Chest. 1988;93(2):318–24. 3338299 10.1378/chest.93.2.318

[8] Thompson DA, Makary MA, Dorman T, Pronovost PJ, Thompson DA, Makary MA, et al Clinical and economic outcomes of hospital acquired pneumonia in intra-abdominal surgery patients. Annals of Surgery. 2006;243(4):547–52. 16552208 10.1097/01.sla.0000207097.38963.3b PMC1448956

[9] Leu HS, Kaiser DL, Mori M, Woolson RF, Wenzel RP. Hospital-acquired pneumonia. Attributable mortality and morbidity. American Journal of Epidemiology. 1989;129(6):1258–67. 2729261 10.1093/oxfordjournals.aje.a115245

[10] Mylotte JM, Graham R, Kahler L, Young BL, Goodnough S. Impact of nosocomial infection on length of stay and functionnal improvement among patients admitted to an acute rehabilitation unit. Infection Control and Hospital Epidemiology. 2001;22(2):83–7. 11232883 10.1086/501868

[11] Heo SM, Haase EM, Lesse AJ, Gill SR, Scannapieco FA, Heo S-M, et al Genetic relationships between respiratory pathogens isolated from dental plaque and bronchoalveolar lavage fluid from patients in the intensive care unit undergoing mechanical ventilation. Clinical Infectious Diseases. 2008;47(12):1562–70. 10.1086/593193 18991508 PMC3582026

[12] Terpenning MS, Taylor GW, Lopatin DE, Kerr CK, Dominguez L, Loesche WJ. Aspiration pneumonia: dental and oral risk factors in an older veteran population. Journal of the American Geriatrics Society. 2001;49(5):557–63. 11380747 10.1046/j.1532-5415.2001.49113.x

[13] Fourrier F, Cau-Pottier E, Boutigny H, Roussel-Delvallez M, Jourdain M, Chopin C. Effects of dental plaque antiseptic decontamination on bacterial colonization and nosocomial infections in critically ill patients. Intensive Care Medicine. 2000;26(9):1239–47. 11089748 10.1007/s001340000585

[14] Scannapieco FA, Stewart EM, Mylotte JM. Colonization of dental plaque by respiratory pathogens in medical intensive care patients. Critical Care Medicine. 1992;20(6):740–5. 1597025 10.1097/00003246-199206000-00007

[15] El-Solh AA, Pietrantoni C, Bhat A, Okada M, Zambon J, Aquilina A, et al Colonization of dental plaques: a reservoir of respiratory pathogens for hospital-acquired pneumonia in institutionalized elders.[see comment]. Chest. 2004;126(5):1575–82. 15539730 10.1378/chest.126.5.1575

[16] Fourrier F, Duvivier B, Boutigny H, Roussel-Delvallez M, Chopin C. Colonization of dental plaque: a source of nosocomial infections in intensive care unit patients.[see comment]. Critical Care Medicine. 1998;26(2):301–8. 9468169 10.1097/00003246-199802000-00032

[17] Chan EY, Ruest A, Meade MO, Cook DJ. Oral decontamination for prevention of pneumonia in mechanically ventilated adults: systematic review and meta-analysis.[see comment]. BMJ. 2007;334(7599):889 17387118 10.1136/bmj.39136.528160.BE PMC1857782

[18] Chlebicki MP, Safdar N. Topical chlorhexidine for prevention of ventilator-associated pneumonia: a meta-analysis. Critical Care Medicine. 2007;35(2):595–602. 17205028 10.1097/01.CCM.0000253395.70708.AC

[19] Morris AC, Hay AW, Swann DG, Everingham K, McCulloch C, McNulty J, et al Reducing ventilator-associated pneumonia in intensive care: impact of implementing a care bundle. Critical Care Medicine. 2011;39(10):2218–24. 10.1097/CCM.0b013e3182227d52 21666444

[20] Yoneyama T, Yoshida M, Ohrui T, Mukaiyama H, Okamoto H, Hoshiba K, et al Oral care reduces pneumonia in older patients in nursing homes.[see comment]. Journal of the American Geriatrics Society. 2002;50(3):430–3. 11943036 10.1046/j.1532-5415.2002.50106.x

[21] Bassim CW, Gibson G, Ward T, Paphides BM, Denucci DJ, Bassim CW, et al Modification of the risk of mortality from pneumonia with oral hygiene care. Journal of the American Geriatrics Society. 2008;56(9):1601–7. 10.1111/j.1532-5415.2008.01825.x 18691286

[22] Quinn B, Baker DL, Cohen S, Stewart JL, Lima CA, Parise C. Basic nursing care to prevent nonventilator hospital-acquired pneumonia. Journal of Nursing Scholarship. 2014;46(1):11–9. 10.1111/jnu.12050 24119253

[23] Binkley CJ, Haugh GS, Kitchens DH, Wallace DL, Sessler DI. Oral microbial and respiratory status of persons with mental retardation/intellectual and developmental disability: an observational cohort study. Oral Surgery, Oral Medicine, Oral Pathology, Oral Radiology and Endodontology. 2009;108(5):722–31. 10.1016/j.tripleo.2009.06.027 PMC2763931 19748295

[24] Ewan V, Perry JD, Mawson T, McCracken G, Brown AN, Newton J, et al Detecting potential respiratory pathogens in the mouths of older people in hospital. Age & Ageing. 2010;39(1):122–5. 19749149 10.1093/ageing/afp166 PMC2794360

[25] Kollef MH, Shorr A, Tabak YP, Gupta V, Liu LZ, Johannes RS. Epidemiology and outcomes of health-care-associated pneumonia: results from a large US database of culture-positive pneumonia.[see comment][erratum appears in Chest. 2006 Mar;129(3):831]. Chest. 2005;128(6):3854–62. 16354854 10.1378/chest.128.6.3854

[26] Fourrier F, Dubois D, Pronnier P, Herbecq P, Leroy O, Desmettre T, et al Effect of gingival and dental plaque antiseptic decontamination on nosocomial infections acquired in the intensive care unit: a double-blind placebo-controlled multicenter study.[see comment]. Critical Care Medicine. 2005;33(8):1728–35. 16096449 10.1097/01.ccm.0000171537.03493.b0

[27] Scannapieco FA, Yu J, Raghavendran K, Vacanti A, Owens SI, Wood K, et al A randomized trial of chlorhexidine gluconate on oral bacterial pathogens in mechanically ventilated patients. Critical care (London, England). 2009;13(4):R117 10.1186/cc7967 19765321 PMC2750165

[28] Hanson LC, Weber DJ, Rutala WA. Risk factors for nosocomial pneumonia in the elderly. American Journal of Medicine. 1992;92(2):161–6. 1543200 10.1016/0002-9343(92)90107-m

[29] Terpenning MS, Taylor GW, Lopatin DE, Kerr CK, Dominguez BL, Loesche WJ. Aspiration pneumonia: dental and oral risk factors in an older veteran population.[see comment]. Journal of the American Geriatrics Society. 2001;49(5):557–63. 11380747 10.1046/j.1532-5415.2001.49113.x

[30] Belda J, Cavalcanti M, Ferrer M, Serra M, Puig de la Bellacasa J, Canalis E, et al Bronchial colonization and postoperative respiratory infections in patients undergoing lung cancer surgery. Chest. 2005;128(3):1571–9. 16162760 10.1378/chest.128.3.1571

[31] Dilworth JP, White RJ, Brown EM. Oropharyngeal flora and chest infection after upper abdominal surgery. Thorax. 1991;46(3):165–7. 2028430 10.1136/thx.46.3.165 PMC463018

[32] Demidenko E. Sample size determination for logistic regression revisited. Statistics in Medicine.26(18):3385–97. 17149799 10.1002/sim.2771

[33] Russell SL, Boylan RJ, Kaslick RS, Scannapieco FA, Katz RV. Respiratory pathogen colonization of the dental plaque of institutionalized elders. Special Care in Dentistry. 1999;19(3):128–34. 10860077 10.1111/j.1754-4505.1999.tb01413.x

[34] Preston AJ, Gosney MA, Noon S, Martin MV. Oral flora of elderly patients following acute medical admission. Gerontology. 1999;45(1):49–52. 9852381 10.1159/000022055

[35] Mahoney FI, Barthel DW. Functional Evaluation: The Barthel Index. Maryland State Medical Journal. 1965;14:61–5. 14258950

[36] Rockwood K, Song X, MacKnight C, Bergman H, Hogan DB, McDowell I, et al A global clinical measure of fitness and frailty in elderly people. CMAJ Canadian Medical Association Journal. 2005;173(5):489–95. 16129869 10.1503/cmaj.050051 PMC1188185

[37] Rockwood K, Rockwood MRH, Andrew MK, Mitnitski A. Reliability of the hierarchical assessment of balance and mobility in frail older adults. Journal of the American Geriatrics Society. 2008;56(7):1213–7. 10.1111/j.1532-5415.2008.01773.x 18503518

[38] Turesky S, Gilmore ND, Glickman I. Reduced plaque formation by the chloromethyl analogue of victamine C. Journal of Periodontology. 1970;41(1):41–3. 5264376 10.1902/jop.1970.41.41.41

[39] Quigley GA, Hein JW. Comparative cleansing efficiency of manual and power brushing. Journal of the American Dental Association. 1962;65:26–9. 14489483 10.14219/jada.archive.1962.0184

[40] American Thoracic Society, Infectious Diseases Society of A, American Thoracic S, Infectious Diseases Society of A. Guidelines for the management of adults with hospital-acquired, ventilator-associated, and healthcare-associated pneumonia.[see comment]. American Journal of Respiratory & Critical Care Medicine. 2005;171(4):388–416. 15699079 10.1164/rccm.200405-644ST

[41] Masterton RG, Galloway A, French G, Street M, Armstrong J, Brown E, et al Guidelines for the management of hospital-acquired pneumonia in the UK: Report of the working party on hospital-acquired pneumonia of the british society for antimicrobial chemotherapy. Journal of Antimicrobial Chemotherapy. 2008;62(1):5–34. 10.1093/jac/dkn162 18445577 PMC7110234

[42] Griscelli F, Barrois M, Chauvin S, Lastere S, Bellet D, Bourhis JH. Quantification of human cytomegalovirus DNA in bone marrow transplant recipients by real-time PCR. Journal of Clinical Microbiology. 2001;39(12):4362–9. 11724846 10.1128/JCM.39.12.4362-4369.2001 PMC88550

[43] Saeed K, Ahmad N, Pallett A, Guiver M, Marsh P. Specific staphylococcal polymerase chain reaction can be a complementary tool for identifying causative organisms and guiding antibiotic management in orthopaedic infections. Current Orthopaedic Practice. 2010;21(6):628–31.

[44] Mojon P, Budtz-Jorgensen E, Michel JP, Limeback H. Oral health and history of respiratory tract infection in frail institutionalised elders. Gerodontology. 1997;14(1):9–16. 9610298 10.1111/j.1741-2358.1997.00009.x

[45] Brook I. The impact of smoking on oral and nasopharyngeal bacterial flora. Journal of Dental Research. 2011;90(6):704–10. 10.1177/0022034510391794 21558542

[46] Nicolle LE, McLeod J, McIntyre M, MacDonell JA. Significance of pharyngeal colonization with aerobic gram-negative bacilli in elderly institutionalized men. Age and Ageing. 1986;15(1):47–52. 3082122 10.1093/ageing/15.1.47

[47] Sumi Y, Kagami H, Ohtsuka Y, Kakinoki Y, Haruguchi Y, Miyamoto H. High correlation between the bacterial species in denture plaque and pharyngeal microflora. Gerodontology. 2003;20(2):84–7. 14697018 10.1111/j.1741-2358.2003.00084.x

[48] Freemantle N, Richardson M, Wood J, Ray D, Khosla S, Shahian D, et al Weekend hospitalization and additional risk of death: An analysis of inpatient data. Journal of the Royal Society of Medicine. 2012;105(2):74–84. 10.1258/jrsm.2012.120009 22307037 PMC3284293

[49] Berdal JE, Bjornholt J, Blomfeldt A, Smith-Erichsen N, Bukholm G. Patterns and dynamics of airway colonisation in mechanically-ventilated patients. Clinical Microbiology & Infection. 2007;13(5):476–80. 17430338 10.1111/j.1469-0691.2006.01678.x

[50] Ewig S, Torres A, El-Ebiary M, Fabregas N, Hernandez C, Gonzalez J, et al Bacterial colonization patterns in mechanically ventilated patients with traumatic and medical head injury. Incidence, risk factors, and association with ventilator-associated pneumonia. American Journal of Respiratory & Critical Care Medicine. 1999;159(1):188–98. 9872838 10.1164/ajrccm.159.1.9803097

[51] Mobbs KJ, van Saene HK, Sunderland D, Davies PD. Oropharyngeal gram-negative bacillary carriage in chronic obstructive pulmonary disease: relation to severity of disease. Respiratory Medicine. 1999;93(8):540–5. 10542986 10.1016/s0954-6111(99)90152-x

[52] Niederman MS. Gram-negative colonization of the respiratory tract: pathogenesis and clinical consequences. Seminars in Respiratory Infections. 1990;5(3):173–84. 2255803

[53] Palmer LB, Albulak K, Fields S, Filkin AM, Simon S, Smaldone GC. Oral clearance and pathogenic oropharyngeal colonization in the elderly. American Journal of Respiratory & Critical Care Medicine. 2001;164(3):464–8. 11500351 10.1164/ajrccm.164.3.2008149

[54] Dickson RP, Erb-Downward JR, Huffnagle GB. Towards an ecology of the lung: new conceptual models of pulmonary microbiology and pneumonia pathogenesis. The Lancet Respiratory Medicine. 2014;2(3):238–46. 10.1016/S2213-2600(14)70028-1 24621685 PMC4004084

[55] Morris AC, Kefala K, Simpson AJ, Wilkinson TS, Everingham K, Kerslake D, et al Evaluation of the effect of diagnostic methodology on the reported incidence of ventilator-associated pneumonia. Thorax. 2009;64(6):516–22. 10.1136/thx.2008.110239 19213771

[56] Lieberman D, Shleyfer E, Castel H, Terry A, Harman-Boehm I, Delgado J, et al Nasopharyngeal versus oropharyngeal sampling for isolation of potential respiratory pathogens in adults. Journal of Clinical Microbiology. 2006;44(2):525–8. 16455908 10.1128/JCM.44.2.525-528.2006 PMC1392694

