# Supplementary material for: Dental and Microbiological Risk Factors for Hospital-Acquired Pneumonia in Non-Ventilated Older Patients
Source: PLoS One. 2015 Apr 29;10(4):e0123622. doi: 10.1371/journal.pone.0123622 (PMC4414413; doi:10.1371/journal.pone.0123622)
Supplement: S3 Dataset — (DOCX) [file pone.0123622.s003.docx]

Codes for Oral Flora Database

**Teeth**

1=teeth

2= dentures only

3=teeth and dentures

Dentures

1=no dentures

2=upper dentures

3=lower dentures

4=upper and lower dentures

Anaesthetic

1=GA, LMA, Femoral nerve block

2=Spinal

3= GA ETT Femoral nerve block

4=GA ETT

Anaesthetic2

1= GA ETT Femoral nerve block

2= GA ETT

3= GA, LMA, Femoral nerve block

4= Spinal

Cough

1=no cough

2=dry cough

3=rattly unproductive

4=productive

Sputum

1=none

2= clear

3=white

4=yellow/green

**Oxygen**

1=none

2= 2l per nasal

3=24%

4=4l per nasal

5=28%

6=35%

7=40%

8=60%

9=100%

Residence pre admit

1= own home

4=residential

5=nursing home

6=Hospital

Smoking

1=current

2=ex

3=never
