# Supplementary material for: Dental and Microbiological Risk Factors for Hospital-Acquired Pneumonia in Non-Ventilated Older Patients
Source: PLoS One. 2015 Apr 29;10(4):e0123622. doi: 10.1371/journal.pone.0123622 (PMC4414413; doi:10.1371/journal.pone.0123622)
Supplement: S1 Table — (DOCX) [file pone.0123622.s004.docx]

| Target organism | Gene target | Primer sequences | Primer concentrations µM(f/r) | Probe sequence and dye label (5’-3’) | *Probe concentration* *µM* |
| --- | --- | --- | --- | --- | --- |
| *S. aureus* [[43](#_ENREF_43)] | femB | F GAC ATT TGA TAG TCA ACG TAA ACG TAA ACG TAA TAT T  R GCT CTT CAG TTT CAC GAT ATA AAT CTA AGA | 3/3 | **VIC**- TCA TCA CGT TCA AGG AAT CTG ACT TTA ACA CCA TAG T- **TAMRA** | 2 |
| MRSA[[43](#_ENREF_43)] | mecA | F CAT TGA TCG CAA CGT TCA ATT T  R TGG TCT TTC TGC ATT CCT GGA | 1/1 | **CY5**-TGG AAG TTA GAT TGG GAT CAT AGC GTC AT- **DDQII** | 1 |
| *E. coli* | uidA | F CGC GCT TTC CCA CCA A  R CGG CCT GTG GGC ATT C | 0.9/0.9 | **CY5**-CAA TTC CAC AGT TTT CGC GAT CCA GAC- **DDQII** | 0.25 |
| *P. aeruginosa* | ecfX | F GCC TGT CCC AGG TCG AAG T  R GAT GTG CTT TTC CAC CAT GCT | 0.05/0.9 | **VIC**-CCG AGC GCA TGG GAA TCT CCC- **TAMRA** | 0.25 |
| *S. pneumoniae* | cps | F GTG TCG CTG TTT TAG CAG ATA GTG A  R TCC CAG TCG GTG CTG TCA | 0.3/0.3 | **VIC-** AAA ATG TTA CGC AAC TGA C-**MGB** | 0.25 |
| *H. influenzae* | P6 | F AAA CGG TAT TGT AAC GTT GTT GAA GA  R CAG GTT CTG TAG CTG CAT TAG CA | 0.9/09 | **FAM**-CAG CAA CAG AGT AAC CGC CAA AAG TTT GA- **BHQ1** | 0.25 |
| *Acinetobacter* spp | 16s | F TCA GAC CCA CCA TGA CTT TGA C  R GGT GGA GAC TAG GAG AGT CGA ACT | 0.9/0.9 | **CY5**-TAG AGC GCC TGC TTT GCA CGC A-**DDQII** | 0.25 |
| Human cells[[42](#_ENREF_42)] | GAPDH | F CTC CCC ACA CAC ATG CAC TTA  R CCT AGT CCC AGG GCT TTG ATT | 0.4/0.4 | **VIC**- AAA AGA GCT AGG AGG GAC AGG CAA CTT GGC **TAMRA** | 0.1 |

Abbreviations: f=Forward r=Reverse,
