# Supplementary material for: Dental and Microbiological Risk Factors for Hospital-Acquired Pneumonia in Non-Ventilated Older Patients
Source: PLoS One. 2015 Apr 29;10(4):e0123622. doi: 10.1371/journal.pone.0123622 (PMC4414413; doi:10.1371/journal.pone.0123622)
Supplement: S2 Table — (DOCX) [file pone.0123622.s005.docx]

| Organism | Positive risks | Negative risks |
| --- | --- | --- |
| *E. coli* | No dentures* Male*** Current smoking*** | Few teeth** Barthel index (more frail)*** |
| *S. pneumoniae* | Female* More teeth*** Current smoking*** | Clinical frailty score (less frail)* Charlson index (fewer serious illnesses)* Deprivation score (less deprived) |
| *H. influenzae* | Dentures (complete) ** Female**  Deprivation score (more deprived)** | Age (younger)** Barthel score (more frail)*** |
| *S. aureus* | Charlson index** Admission plaque (higher)*  Barthel index* Male**  Current smoking** | Low deprivation score |
| MRSA | Age* Charlson index*  Current or ex smoker* Barthel index (less frail)* | N/A |

*p<0.05, **p<0.01, ***p<0.001 Note: The final medical models for *E. coli* and *Acinetobacter* were underdispersed, and are not shown. No significant associations were found for *P. aeruginosa* (either model) or *Acinetobacter* spp (dental model).
